# The mystery of the butterfly bush *Buddleja davidii*: How are the butterflies attracted?

**DOI:** 10.3389/fpls.2022.994851

**Published:** 2022-09-02

**Authors:** Simon Lehner, Stefan Schulz, Stefan Dötterl

**Affiliations:** ^1^Department of Environment and Biodiversity, Paris-Lodron University of Salzburg, Salzburg, Austria; ^2^Institute of Organic Chemistry, Technische Universität Braunschweig, Braunschweig, Germany

**Keywords:** butterfly pollination syndrome, chemical communication, nectar host, oxoisophorones, peacock butterfly, visual and olfactory signals

## Abstract

Many plant species are pollinated by butterflies. These insects are primarily attracted by visual flower cues, however, butterflies are also known to respond to flower scents and some butterfly-pollinated plants are strongly scented. One of such plants is the butterfly bush, *Buddleja davidii*, which is a magnet for butterflies. It is widespread in its native region in Asia and famous for its success in invasive spreading in regions throughout the world. Due to its attractiveness to butterflies and its beautiful and conspicuous inflorescences, it also is an important ornamental, found in many gardens. Here, we elucidated the signaling between the butterfly bush and one of its abundant visitors, the peacock butterfly (*Aglais io*), using chemical and behavioral approaches. We found that olfactory cues are more attractive than visual cues, and that feeding behavior is only elicited by olfactory cues, most effectively by 4-oxoisophorone and oxoisophorone epoxide. The latter compound was not known to elicit behavioral responses in pollinators before this study. The relative importance of olfactory cues was higher in our study than previously observed in any butterfly pollination system. The identified attractants might contribute to the widespread occurrence of the butterfly bush in its native region in Asia and its success in invasive spreading in regions throughout the world.

## Introduction

The majority of flowering plants is pollinated by insects and other animals (Ollerton et al., 2011), and plants evolved highly diverse strategies to advertise their flowers, resulting in flowers with various phenotypes (e.g., structure, color, fragrance). Most important for pollinator attraction are visual and olfactory floral cues (Chittka and Thompson, 2001), with their relative importance varying among pollination systems. Nocturnally pollinated plants are known to often rely on olfactory cues to attract their pollinators (e.g., moths, bats; Dobson, 2006), though visual cues might also be involved in such systems, especially in eliciting landing and feeding responses of the animals (Gottsberger and Silberbauer-Gottsberger, 1991; Raguso and Willis, 2002). Olfactory cues are also in some diurnally pollinated plants most responsible for pollinator (e.g., specialized oil bees) attraction (Dötterl et al., 2011), whereas in others mainly visual cues attract their pollinators (e.g., some butterflies; Ômura and Honda, 2005). The relative importance of visual and olfactory cues does not only vary among pollination systems, but, within a system, it also depends on the experience of the flower visitor individuals (e.g., Dötterl et al., 2011).

Bees and flies are the most important pollinators when it comes to the number of plants they pollinate; Lepidoptera, however, are by far the most species rich pollinators. This is especially due to the high number of moth species, but even butterflies alone are more species-rich than bees (Ollerton, 2017). Plants pollinated by butterflies often have conspicuously colored (e.g., red, orange, yellow) flowers and weak to quite strong scents (Willmer, 2011). These scents often consist of compounds of various classes, such as aromatics (e.g., phenylacetaldehyde) and terpenoids (e.g., linalool, oxoisophorones; Dobson, 2006). Though visual floral cues are often more important than olfactory cues for host plant finding in butterflies (Andersson and Dobson, 2003; Dobson, 2006; Hirota et al., 2012; Kinoshita et al., 2017; Barragán-Fonseca et al., 2020; Chen et al., 2021), there are also data, which show that natural and synthetic floral scents elicit strong behavioral responses in butterflies (Honda et al., 1998; Ômura et al., 1999; Andersson, 2003b; Ômura and Honda, 2005), and that synthetic floral scents increase the attractiveness of visual cues (e.g., Ômura and Honda, 2005; Kiepiel and Johnson, 2021).

*Buddleja davidii* Franch. (Scrophulariaceae) is a “magnet” for various butterfly species (e.g., *Aglais io* (L.), *Melanargia galathea* (L.), *Danaus plexippus* (L.), *Papilio machaon* L.) and is, thus, called butterfly bush (Tallent-Halsell and Watt, 2009). Originally native to Asia, *B. davidii* was introduced as ornamental plant to various other parts of the world (e.g., Americas, Europe, New Zealand), where it has often naturalized and become invasive (Tallent-Halsell and Watt, 2009). Besides butterflies, which heavily visit the plant in both native and non-native regions, its flowers are visited, e.g., by moths, wasps, beetles and hummingbirds (Guédot et al., 2008; Tallent-Halsell and Watt, 2009). *Buddleja davidii* is an obligate outcrosser and the high attractiveness of its flowers to pollinators is believed to explain its large native distribution (Chen et al., 2011) and to contribute to its invasive spread in non-native regions (Ebeling et al., 2012). The flowers are typically purple or lilac, have an orange nectar guide and release a strong, pleasant scent (Tallent-Halsell and Watt, 2009; Ebeling et al., 2012; Chen et al., 2014). Abundant compounds are, among others, the irregular terpenoid 4-oxoisophorone and derivatives thereof, the sesquiterpene (*E*,*E*)-α-farnesene and the aromatic compound 2-phenylethanol (Schulz et al., 1988; Andersson et al., 2002; Andersson, 2003a; Chen et al., 2014). Many of the compounds released by the flowers elicit physiological responses in the antennae of nymphalid and pierid butterflies (Andersson, 2003a) indicating that these butterflies might use these compounds to locate the flowers. Indeed, some of the compounds detected in scent samples of *B. davidii* are also released from other plants visited by butterflies, where they were shown to elicit approaching and feeding behaviors (e.g., 2-phenylethanol, phenylacetaldehyde; Honda et al., 1998; Ômura et al., 1999; Ômura and Honda, 2005). However, neither is the relative importance of visual and olfactory floral cues of *B. davidii* in attracting butterflies known, nor has the effect of the abundant compounds in the scent (e.g., oxoisophorone epoxide) on the behavioral activity of the butterflies been studied so far.

Here, we elucidated the signaling between *B. davidii* and the peacock butterfly, *A. io*, one of its abundant visitors in Europe (Tallent-Halsell and Watt, 2009), to identify the cues responsible for the strong attractiveness of this plant to butterflies. Specifically, we (1) investigated the relative importance of visual and olfactory cues for attracting *B. davidii*-naive butterflies and how the search image is shaped through learning in attracting *B. davidii*-experienced individuals. For *B. davidii*-naive butterflies, we (2) also measured the response strength to the different stimuli and identified the compounds most responsible for eliciting feeding behavior. We found that the high attractiveness of *B. davidii* to butterflies is mainly due to its inflorescence scent, and that only olfactory cues, especially 4-oxoisophorone and oxoisophorone epoxide, are capable of eliciting feeding behavior in *A. io*.

## Materials and methods

### Study animals

The peacock butterfly, *Aglais io* (L.), a nymphalid, was used for our experiments. Larvae and eggs were collected in Salzburg and bred in a 60 cm x 60 cm x 60 cm dome net cage. They were kept indoors at a temperature of approximately 21°C and fed on potted *Urtica dioica*, the natural primary host plant of *A. io* larvae. The adults that hatched were our 1^st^ generation animals and were released into the flight cage, in which the behavioral experiments were performed (see “Bioassays”). In this cage, they were offered *U. dioica* plants for reproducing, to result in a second generation of animals. To be able to identify the individual butterflies, they were individually marked with color codes on the back side of each wing, using nail polish (Maybelline Jade).

### Bioassays

Behavioral experiments were performed in a flight cage (8 m × 4 m × 2.2 m; Rachersberger et al., 2019). Some potted plants (*Helianthus annuus*, *Achillea* sp., *Campanula persicifolia, C. trachelium, Reseda alba, Ranunculus acris, Salvia sp.*), that served as nectar and pollen source for bees also housed in the cage, were present and partly visited by *A. io*. This setup simulated a natural situation, in which various flowering plants are available. As an additional food source, six sponges in plastic cups with a sucrose-water mixture (1/4; *v*/*v*) were positioned at various locations inside the flight cage. Depending on the experiment performed, branches of *B. davidii* with inflorescences in full bloom were (to obtain *B. davidii*-experienced butterflies) or were not (for *B. davidii*-naive butterflies) additionally available to the butterflies. If available, they were heavily visited by the butterflies.

#### Relative importance of visual and olfactory cues of *Buddleja davidii* in attracting *Aglais io*

**Figure 1:**
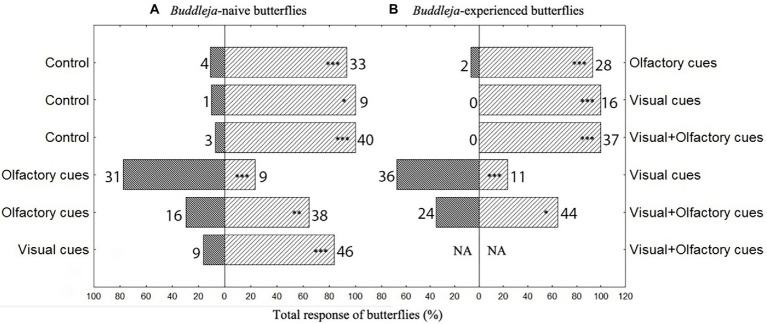
Behavioral responses of *Buddleja davidii*-naive **(A)** as well as experienced **(B)** *Aglais io* butterflies to decoupled visual and olfactory cues or a combination of both cues of *B. davidii*. The responses to the cues presented at the left and the right hand sides of the graph are given in the dark and bright bars, respectively. The numberss next to the bars give absolute numbers of butterflies ^***^p < 0.001, ^**^p < 0.01, ^*^p < 0.05. NA: no data recorded.

Six different dual-choice assays were performed with different kinds of quartz glass cylinders (diameter: 10 cm, height: 29 cm; the same as described in Burger et al., 2010; Dötterl et al., 2011; Milet-Pinheiro et al., 2012) to determine the relative importance of visual and olfactory cues of *B. davidii* in attracting *A. io* (see Figure 1): negative control cylinders were tested against decoupled visual, decoupled olfactory and combined cues; decoupled cues were tested against each other and against combined cues. Each cylinder consisted of a quartz glass cap and body, and a sleeve of Macrolon^®^, which connected and sealed the cap and body. Three to four inflorescences (depending on the number of florets) with approximately 600 florets of *B. davidii*, from the same plant individuals as used for the chemical analyses (see Supplementary material), were placed inside a cylinder. Empty cylinders identical to the cylinders containing the tested inflorescence cues served as negative controls. Depending on the cue tested, the Macrolon^®^ sleeve did (olfactory cues; olfactory + visual cues) or did not (visual cues) have small holes to allow diffusion of inflorescence scents. The cylinders were either transparent (visual, olfactory + visual cues) or painted black with semi-matte varnish (olfactory cues). The cylinders were mounted on a black PVC disk (diameter 11 cm), which was attached to a square wooden table. For each dual-choice assay, the two cylinders were offered 30 cm apart. Between 30 and 40 individuals of the 170–210 *A. io* individuals present in the cage were active (flying around) during the tests.

Each dual-choice assay was repeated three times (3 × 15 min), with the data of the three replicates pooled for data analyses. The position of the cylinders was switched after every replicate to consider possible spatial preferences of the butterflies. All experiments were first performed with *B. davidii*-naive butterflies, then branches of *B. davidii* with inflorescences in full bloom offered to the butterflies, and subsequently the experiments repeated, now with *B. davidii*-experienced butterflies. No distinction between female and male individuals was made as a previous study did not detect differences between genders of *A. io* when searching for nectar resources (Andersson, 2003b). Tests took place between 9 am and 4 pm, when the activity of the animals was high.

Approaches, i.e., flying within 15 cm of the cylinder with a clear change of direction and/or speed, were recorded for both *Buddleja*-naive and -experienced butterflies, while landing on the cylinder and feeding behavior (extension of proboscis, following landing on a cylinder) were additionally recorded for *B. davidii*-naive individuals. Butterflies responding during a 15 min testing period were caught with an insect net, their color code was noted, and they were released back into the cage at the end of the test run. If a butterfly responded more than once in replicate runs of a specific dual-choice assay, only its first response was considered.

#### Effectiveness of different mixtures of compounds and of single oxoisophorones in eliciting feeding behavior in *Buddleja davidii*-naive *Aglais io*

Given that olfactory cues were found to be strongly attractive to the butterflies and that most of the butterflies not only approached the cylinders, but also landed on them and extended their proboscides to look for food (see “Results”), we identified the compounds eliciting the feeding responses in *A. io* (extension of the proboscis; PER). To do so, we used compounds previously described as being electrophysiologically active in gas chromatographic and electroantennographic detections with antennae of *A. io* and scents of *B. davidii* (Andersson, 2003a). Of the 17 EAD-active compounds, 12 were available for our experiments (Supplementary material; see also Supplementary Figure S2). We offered three different compound mixtures (oxoisophorones: 4-oxoisophorone, oxoisophorone epoxide; other terpenes: β-cyclocitral, farnesene, geranylacetone, (*E*)-β-ocimene; and aromatic compounds: benzaldehyde, benzyl alcohol, (*E*)-cinnamic alcohol, (*E*)-cinnamic aldehyde, phenylacetaldehyde, 2-phenylethanol) and the two single compounds of the most effective mixture (oxoisophorones: 4-oxoisophorne, oxoisophorone epoxide; see Results) in petri dishes to the butterflies. Acetone, which was used to dilute the compounds (see Supplementary material), served as negative control. To ensure that the quantitative and relative amounts of these compounds where comparable to the natural scent bouquet of *B. davidii*, we collected dynamic headspace scent samples from petri dishes and inflorescences of *B. davidii*, and analysed them on a GC/MS (gas chromatography/mass spectrometry) system as described previously (Braunschmid et al., 2017; Zito et al., 2019) and in the Supplementary material.

Experiments were also performed in the flight cage, but using plastic water bottles (1.5 l; see Supplementary Figure S3; one bottle per stimulus), into which single butterflies were introduced. After having removed the bottom of these bottles, they were cleaned with ethanol and dried for 2 h at room temperature. As butterflies had difficulties to crawl on the plastic surface, cellulose tissue was placed inside the bottle. A glass petri dish (diameter of 10 cm) with 100 μl of test solution, renewed for each butterfly, was placed onto this tissue. A butterfly was released into the bottle, and the bottle closed at the bottom using another cellulose tissue. The behavior of the butterfly was observed for 5 min. It was recorded whether it elicited the proboscis, and if yes, for how long. 30 *B. davidii-*naive butterflies were first tested on the acetone control, then on the aromatics, the oxoisophorones and finally the other terpenes. 30 different *B. davidii-*naive butterflies were tested on the single compounds, at first on 4-oxoisophorne and then on oxoisophorone epoxide.

### Statistical analyses

To test for differences in butterfly approaches between the cylinders offered in the dual-choice assays, exact binominal tests were performed using the spreadsheet provided at http://www.biostathandbook.com/exactgof.html. The null hypothesis was that the two cylinders are equally attractive to butterflies (e.g., Rachersberger et al., 2019).

Data of the cylinder assays with *B. davidii*-naive butterflies were also used to test for differences among the different stimuli (negative controls, visual cues, olfactory cues, visual + olfactory cues) in their capability to elicit feeding responses. We compared the number of butterflies landing on the cylinders but not extending their proboscides with the number of butterflies that extended their proboscides following the landing responses among the olfactory and olfactory + visual stimuli using a Fisher exact test in STATISTICA (StatSoft Inc., 2012; feeding responses were not elicited by the other stimuli).

Fisher exact tests were also used to test for differences in the likelihood that the *B. davidii*-naive individuals extended their proboscides among the different test substances (solvent control, substance mixtures, single compounds). The null hypothesis in all these tests was that all stimuli had the same attractiveness to the butterflies.

To test for differences in the duration of the proboscis extensions among test stimuli, we used a Kruskal–Wallis ANOVA followed by a non-parametric Tukey HSD *post hoc* test, both provided in STATISTICA, to analyze the experiments with the compound mixtures, and a Mann–Whitney U-test, again in STATISTICA, for analyzing the experiments with oxoisophorone epoxide and 4-oxoisophorone.

## Results

### Relative importance of visual and olfactory cues of *Buddleja davidii* in attracting *Aglais io*

**Figure 2:**
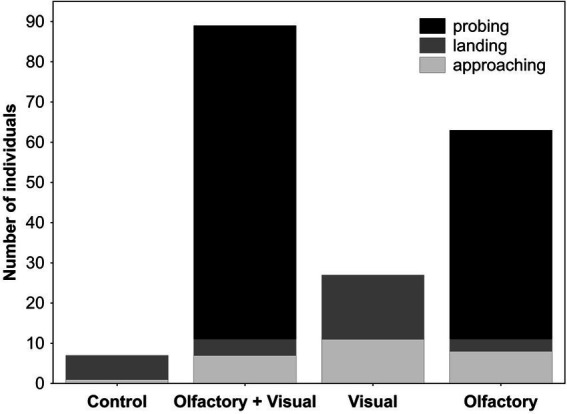
Approaching, landing and probing behaviors of *Buddleja davidii*-naive *Aglais io* butterflies to different cues of flowering branches of *B. davidii*. Probing behavior was only elicited by stimuli that included olfactory cues, whereas olfactory cues alone were as effective as olfactory + visual cues in eliciting this response (Fisher exact test: *p* = 0.19).

*Buddleja davidii*-naive and -experienced butterflies responded very similarly in the dual-choice assays (Figure 1). Independent of their experience with *B. davidii*, visual, olfactory and the combination of both cues all attracted significantly more butterflies than the respective controls. When tested against each other, olfactory cues were more attractive than visual cues. The combined olfactory + visual cues were more attractive than the single cues (Figure 1; visual cues against the combined cues were only tested in *B. davidii*-naive butterflies). Most of the *B. davidii*-naive butterflies that responded in the dual-choice assays to any of the treatments not only approached the cylinders, but also landed on them (Figure 2). The stimuli that included olfactory cues also elicited probing responses, with olfactory cues alone being as effective in eliciting feeding responses as olfactory + visual cues (Fisher exact test: *p* = 0.19).

### Effectiveness of different mixtures of compounds and of single oxoisophorones in eliciting feeding behavior in *Buddleja davidii*-naive *Aglais io*

**Figure 3:**
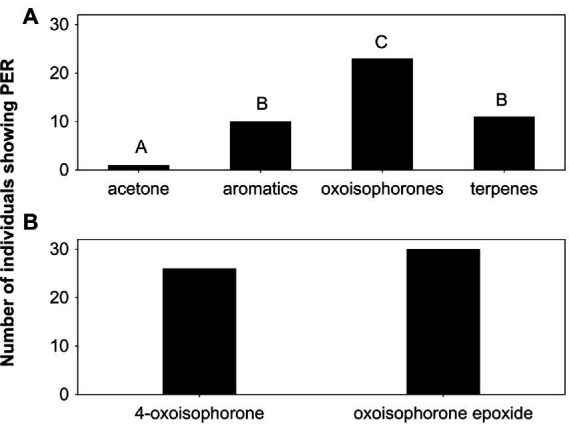
Number of individuals of *Buddleja davidii*-naive *Aglais io* butterflies that extended their proboscides (PER) when tested on **(A)** different compound mixtures and the acetone control, and **(B)** single compounds. Thirty butterflies were tested on the compounds mixtures and acetone, and 30 others on the different single substances. Different letters indicate significant differences in behavioral activity among the different stimuli in **(A)**, whereas the two oxoisophorones were similarly active **(B)**. terpenes: terpenoids other than oxoisophorones.

Compound mixtures comprising aromatics, oxoisophorones, or other terpenes were all more effective in eliciting proboscis extensions in the *B. davidii*-naive butterflies than acetone negative controls, with the oxoisophorones being overall most attractive (global and pairwise Fisher exact tests: *p* ≤ 0.006; Figure 3A). Nearly 80% of the butterflies showed feeding behavior when tested on oxoisophorones, approximately one third when tested on aromatics and on other terpenes, and only 3% when tested on the acetone control. When tested separately, 4-oxoisophorone (87%) and oxoisophorone epoxide (100%) were similarly effective in eliciting feeding behavior in the butterflies (Fisher exact test: *p* = 0.11; Figure 3B).

**Figure 4:**
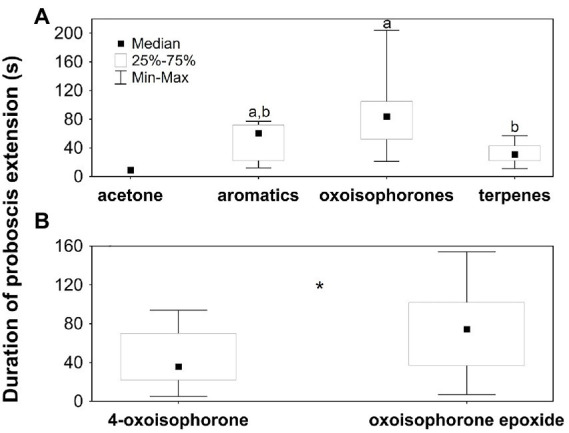
Duration of proboscis extensions of *Buddleja davidii*-naive *Aglais io* butterflies when tested on **(A)** acetone (*N* = 1) and compound mixtures consisting of aromatics (*N* = 10), oxoisophorones (*N* = 23) and terpenes other than oxoisophorones (*N* = 11), and **(B)** 4-oxoisophorone (*N* = 26) and oxoisophorone epoxide (*N* = 30). Only butterflies that extended the proboscis were included in the analysis, as evidenced in Figure 3. In **(A)** different letters indicate significant differences among the different stimuli, excluding the single proboscis extension to acetone. In **(B)** the asterisk (*) indicates a significant difference (*p* = 0.01). terpenes: terpenoids other than oxoisophorones.

The different compound mixtures (excluding acetone; KW-ANOVA: *H*_2;*N* = 44_ = 15.2; *p* = 0.001; Figure 4A) and the individual oxoisophorones (*U*-test: *Z*_*N* = 56_ = −2.8; *p* = 0.01; Figure 4B) also elicited proboscis extensions of different duration (Figure 4). The duration of the proboscis extension was longer in response to oxoisophorones than to the other terpenes and intermediate in response to the aromatic compounds. 4-Oxoisophorone elicited significantly shorter extensions than oxoisophorone epoxide.

## Discussion

Our results demonstrate that visual and olfactory cues of *Buddleja davidii* are attractive to both *B. davidii*-naive and -experienced *Aglais io* butterflies, with the olfactory cues being more attractive than the visual cues. As demonstrated for *B. davidii*-naive individuals, olfactory cues not only attract the butterflies from distance, but, in contrast to visual cues, are required to elicit feeding behavior in *A. io*. We identified oxoisophorone derivatives as most effective in eliciting feeding behavior, when compared to other terpenes and to aromatic compounds.

Our finding that both visual and olfactory cues of host plants are behaviorally active (Figure 1) confirms previous studies (e.g., Ômura and Honda, 2005). However, the relative importance of olfactory cues was higher in our study than previously observed (Kinoshita et al., 2017). This difference is most obvious when comparing the results of the present study with those obtained by Barragán-Fonseca et al. (2020) and Chen et al. (2021), which are the only studies, as far as we know, that also performed choice assays with both natural visual and natural olfactory floral cues. We found that olfactory cues of *B. davidii* are more than three times more attractive to *A. io* than visual cues (Figure 1), while Barragán-Fonseca et al. (2020) recorded an about tenfold higher attractiveness of visual cues compared to olfactory cues of *Brassica nigra* to *Pieris brassicae*, and Chen et al. (2021) registered approaches of *Papilio* butterflies only to visual cues of *Habenaria rhodocheila* (Orchidaceae) when tested against olfactory cues. These differences among the studies might relate to different search images in different butterfly species involved, or, more likely, with differences in the nature of the visual and olfactory cues of the tested plants. Both cues strongly differ between the three plants, with *B. davidii* having smaller but many more flowers in an inflorescence than *B. nigra* and *H. rhodocheila.* Flowers are lilac/purple in *B. davidii,* but yellow and orange in *B. nigra* and *H. rhodocheila*, respectively, while the scents are much stronger and comprise many more components in *B. davidii* than *Brassica nigra* (e.g., Andersson et al., 2002; Barragán-Fonseca et al., 2020), with even no scent detectable to the human nose in *H. rhodocheila* (Chen et al., 2021). Overall, given that butterflies visit plants with various visual and olfactory displays (Andersson et al., 2002; Kinoshita et al., 2017), it seems plausible that the relative importance of visual and olfactory cues strongly varies among systems, as also known for other pollination systems, e.g., between bees and their host plants (Burger et al., 2010; Dötterl et al., 2011). In our study, foraging experience did not have an influence on the relative importance of the different cue modalities, though butterflies are generally capable of learning visual and olfactory floral cues (Andersson and Dobson, 2003; Andersson, 2003b; Kinoshita et al., 2017).

We were surprised by our finding that the feeding behavior of the butterflies (data only for B. *davidii*-naive individuals recorded) was only elicited by olfactory cues of *B. davidii* (Figure 2). This is because other nymphalid butterflies (as shown for flower-naive *Vanessa indica*) are known to extend their proboscides to colored and scentless artificial flowers (Ômura and Honda, 2005). An explanation for these different findings might be that nymphalid butterflies in general (e.g., blue, yellow, orange, red; Kinoshita et al., 2017) and *A. io* in particular (blue, yellow; Kinoshita et al., 2017) have preferences for colors other than purple. Butterflies from the papilionid family that also visit *B. davidii* (Tallent-Halsell and Watt, 2009) might behave differently, as several species in this family have preferences for purple (Kinoshita et al., 2017). Our finding is also in sharp contrast to what is known from nocturnal Lepidoptera (*Manduca sexta*), which only extend their proboscides when both visual and olfactory cues are available (Raguso and Willis, 2002, 2005).

Oxoisophorones were the most effective feeding stimulants of *B. davidii* and induced proboscis extensions in similar frequency as the natural scent, with 4-oxoisophorone and oxoisophorone epoxide being similarly active (Figure 3). This suggests that feeding behavior in *A. io* toward olfactory cues of *B. davidii* is mainly induced by the two oxoisophorones, despite the fact, that mixtures of other terpenes and aromatic compounds also elicited feeding responses when offered separately to *A. io* (Figure 4). Benzaldehyde, phenylacetaldehyde and 2-phenylethanol, aromatic compounds used in the present study, are well known to elicit feeding responses also in other butterflies (Kinoshita et al., 2017, and references therein).

To the best of our knowledge, the behavioral attractiveness of oxoisophorone epoxide to flower visiting insects or other animals has not been reported yet. In contrast, 4-oxoisophorone is a known attractant for insects of different orders (Hymenoptera, Diptera and Lepidoptera, including butterflies; Landolt et al., 2014; El-Sayed et al., 2018; Nagy et al., 2022). 4-Oxoisophorone occurs in various butterfly-pollinated plants of different phylogenetic lineages (Andersson et al., 2002) and also in androconial organs of Danainae (Nymphalidae) butterflies, where it might have a pheromonal function (Schulz et al., 1988). It was suggested to have evolved as floral scent in response to sensory preferences of butterflies for this compound (Andersson et al., 2002), which also might be true for oxoisophorone epoxide, given its high attractiveness to butterflies in the present study. It elicited proboscis extensions of even longer duration than 4-oxoisophorone (Figure 4).

We conclude that the high attractiveness of *B. davidii* for peacock butterflies is due to its visual, but more importantly, its olfactory cues. Only the latter cues elicit feeding behavior besides approaching and landing. Most responsible for eliciting feeding behavior were 4-oxoisophorone and oxoisophorone epoxide, the latter of which we introduce to the literature as a new pollinator attractant. When compared with other butterfly pollinated plants, *B. davidii* releases these two compounds in higher amounts (Andersson et al., 2002), explaining its enormous attractiveness to peacock butterfly pollinators. Whether these chemicals are highly attractive not only for the peacock butterfly but also for other butterfly visitors of *B. davidii,* is topic for potential future studies. Overall, the presence of 4-oxoisophorone and oxoisophorone epoxide in high amounts in its floral scent might contribute to a high pollination success and a prolific seed production (Tallent-Halsell and Watt, 2009), and together with other characteristics of the plant (e.g., short juvenile period, aggressive growth, wide range of tolerances to environmental conditions; Tallent-Halsell and Watt, 2009), be responsible for the widespread occurrence of *B. davidii* in Asia and its success in spreading in regions throughout the world following its introduction.

## Data availability statement

The original contributions presented in the study are included in the article/Supplementary material, further inquiries can be directed to the corresponding authors.

## Author contributions

SD and SL designed the study. SL collected the data. SS provided a synthetic compound. SL and SD analyzed the data and wrote the manuscript to which SS added information about the synthesis of oxoisophorone epoxide and structures of chemicals mentioned in the study. All authors contributed to the article and approved the submitted version.

## Conflict of interest

The authors declare that the research was conducted in the absence of any commercial or financial relationships that could be construed as a potential conflict of interest.

## Publisher’s note

All claims expressed in this article are solely those of the authors and do not necessarily represent those of their affiliated organizations, or those of the publisher, the editors and the reviewers. Any product that may be evaluated in this article, or claim that may be made by its manufacturer, is not guaranteed or endorsed by the publisher.
