# Supplementary figures and images for: The mystery of the butterfly bush Buddleja davidii: How are the butterflies attracted?
Source: Front Plant Sci. 2022 Sep 2;13:994851. doi: 10.3389/fpls.2022.994851 (PMC9478603; doi:10.3389/fpls.2022.994851)

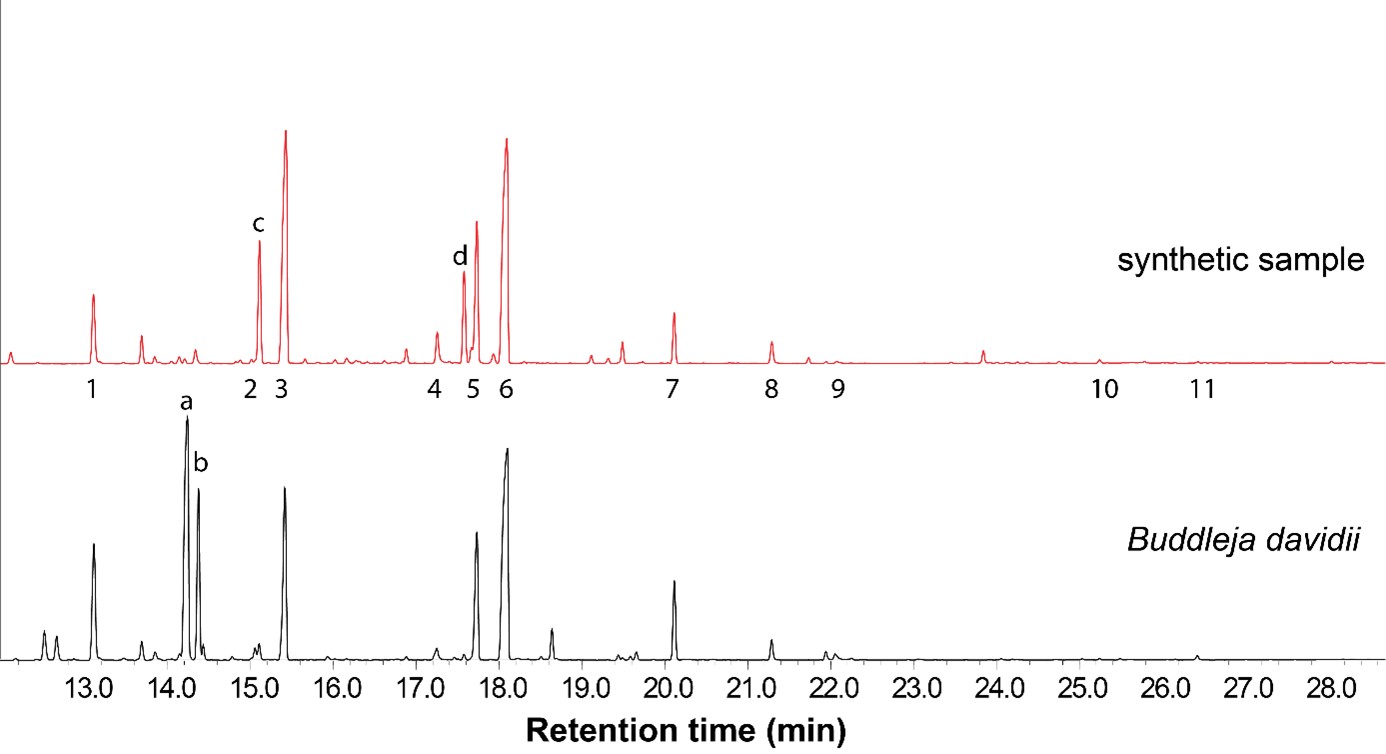

Supplement: Supplementary file 1 [file Image_1.JPEG]

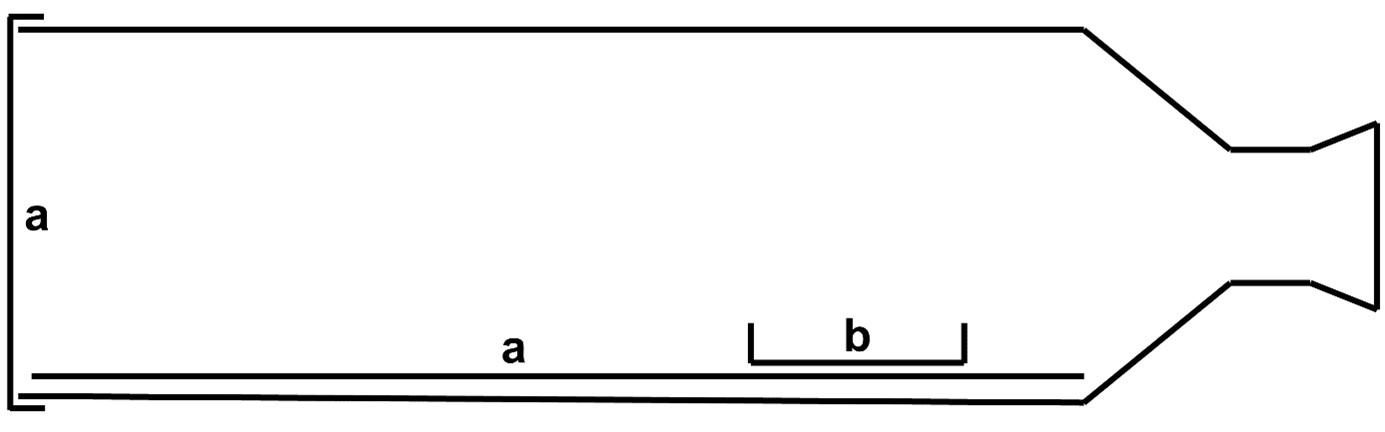

Supplement: Supplementary file 3 [file Image_3.JPEG]
