# Supplementary material for: The mystery of the butterfly bush Buddleja davidii: How are the butterflies attracted?
Source: Front Plant Sci. 2022 Sep 2;13:994851. doi: 10.3389/fpls.2022.994851 (PMC9478603; doi:10.3389/fpls.2022.994851)
Supplement: Supplementary file 4 [file Data_Sheet_1.DOCX]

Supplementary Material

# Supplementary Data

## Chemicals

All chemicals except the irregular terpene oxoisophorone epoxide, which was synthesized in > 95% purity from 4-oxoisophorone by epoxidation with H_2_O_2_ according to a published procedure [34], were from Sigma-Aldrich (Wien, Austria): the aromatic compounds benzaldehyde (>99%), benzyl alcohol (>99%), (*E*)-cinnamic alcohol (98%), (*E*)-cinnamic aldehyde (99%), phenylacetaldehyde (>90%), 2-phenylethanol (99%); the irregular terpenes β-cyclocitral (>90%), geranylacetone (96%), 4-oxoisophorone (98%); the monoterpene (*E*)-β-ocimene (>90%); the sesquiterpene farnesene (mixture of isomers, 50%).

## Volatile sampling and chemical analyses

Volatiles were collected from the *B. davidii* inflorescences (during daytime) and the petri dishes using dynamic headspace methods (Zito et al. 2019; Braunschmid et al. 2017). Cut inflorescences (collected from individuals growing around the Natural Science Faculty Building of the Paris-Lodron-University of Salzburg) and petri dishes were enclosed in polyester oven bags (20 x15 cm, Toppits®, Germany), and their emitted scents were trapped by sucking the air from the bags into adsorbent tubes. The tubes consisted of glass capillaries (length: 8 cm, inner diameter: 2.5 mm) filled with 15 mg Tenax-TA (mesh 60–80) and 15 mg Carbotrap (mesh 20–40; both Supelco). The air was sucked through the tubes for 4 hours using a membrane pump (G12/01 EB, Rietschle Thomas Inc., Puchheim,Germany) driven by a 9V power supply; the flow rate was adjusted to 200 ml/min. As negative controls, empty oven bags were used. Trapped volatiles were eluted from the adsorbent tubes with 75 μl of acetone and stored at -20°C until chemical analyses.

The scent samples were analyzed by GC/MS using a Shimadzu GC-MS-QP2010 Ultra equipped with a AOC-20i auto injector (Shimadzu, Tokyo, Japan) and a ZB-5 fused silica column (5% phenyl polysiloxane; 30 m long, inner diameter 0.32 mm, film thickness 0.25 µm, Phenomenex) as described in Braunschmid et al. (2017). 1 µl of the samples was injected (injection temperature: 220°C; split: 1) and the column flow (carrier gas: helium) was set at 3 ml min^-1^. The oven temperature of the GC was set at 40°C for 1 min, then increased by 10°C/min to 220°C, and held for 2 min. The MS interface and the ion source worked at 220°C and 200°C, respectively. Mass spectra were recorded at 70 eV (EI mode) from *m/z* 30 to 350. GC/MS data were processed using the GCMSolution package, Version 2.72 (Shimadzu Corporation 2012).

After several trials with various concentrations of the specific compounds, we finally found that 100µl of test solutions with the compounds in following concentrations (µL/L; diluted in acetone, Rotisolv, Roth, Germany) resulted in headspace samples comparable to that of 2-3 inflorescences of *B. davidii* (Fig. S1): benzaldehyde (90), benzyl alcohol (1.7), (*E*)-cinnamic alcohol (780), (*E*)-cinnamic aldehyde (10.7), phenylacetaldehyde (1800), 2-phenylethanol (13.4), β-cyclocitral (11.5), geranylacetone (2.7), oxoisophorone epoxide (98), 4-oxoisophorone (450), (*E*)-β-ocimene (300), farnesene (16.1).

# Supplementary Figures and Tables

## Supplementary Figures

**Supplementary Figure 1.** Total ion chromatogram of headspace samples collected from inflorescences of *Buddleja davidii* and a synthetic scent sample used for behavioral assays. Numbers between the two chromatograms indicate targeted compounds. Letters indicate vegetative compounds of *B. davidii* not used for the analyses (a, b) and compounds occurring as “contaminants” in the synthetic scent mixture (c, d). 1: benzaldehyde, 2: benzyl alcohol, 3: phenylacetaldehyde + (*E*)-β-ocimene, 4: 2-phenylethanol, 5: oxoisophorone epoxide, 6: 4-oxoisophorone, 7: β-cyclocitral, 8: (*E*)-cinnamic aldehyde, 9: (*E*)-cinnamic alcohol, 10: geranylacetone, 11: (*E*,*E*)-α-farnesene. a: (*Z*)-3-hexenyl acetate, b: hexyl acetate, c: (*Z*)-β-ocimene, d: allo-ocimene.

**Supplementary Figure 2.** Compounds mentioned in this study. The colored compounds were used as synthetic material in the respective mixtures and additionally as singular compounds (blue).

**Supplementary Figure 3.** Schematic drawing of a plastic water bottle (1.5 L), as used to test for the effectiveness of different compound mixtures and of single oxoisophorones in eliciting feeding behavior in *A. io*. a: cellulose tissue; b: petri dish, into which test solution was offered to a single butterfly introduced into the bottle.
